# A cross-species analysis method to analyze animal models' similarity to human's disease state

**DOI:** 10.1186/1752-0509-6-S3-S18

**Published:** 2012-12-17

**Authors:** Shuhao Yu, Lulu Zheng, Yun Li, Chunyan Li, Chenchen Ma, Yixue Li, Xuan Li, Pei Hao

**Affiliations:** 1 College of Life Science and Biotechnology, Shanghai Jiaotong University, 800 Dongchuan Road, Shanghai 200240, P.R.China; 2 Key Lab of Systems Biology/Key Laboratory of Synthetic Biology, Shanghai Institutes for Biological Sciences, Chinese Academy of Sciences, 320 Yueyang Road, Shanghai 200031, P.R.China; 3 Institut Pasteur of Shanghai, Shanghai Institutes for Biological Sciences, Chinese Academy of Sciences, 320 Yueyang Road, Shanghai 200031, P.R.China; 4 College of Life Science and Technology, Huazhong University of Science and Technology, Wuhan 430074, P.R.China; 5 Shanghai Center for Bioinformation Technology, Shanghai, P.R.China

## Abstract

**Background:**

Animal models are indispensable tools in studying the cause of human diseases and searching for the treatments. The scientific value of an animal model depends on the accurate mimicry of human diseases. The primary goal of the current study was to develop a cross-species method by using the animal models' expression data to evaluate the similarity to human diseases' and assess drug molecules' efficiency in drug research. Therefore, we hoped to reveal that it is feasible and useful to compare gene expression profiles across species in the studies of pathology, toxicology, drug repositioning, and drug action mechanism.

**Results:**

We developed a cross-species analysis method to analyze animal models' similarity to human diseases and effectiveness in drug research by utilizing the existing animal gene expression data in the public database, and mined some meaningful information to help drug research, such as potential drug candidates, possible drug repositioning, side effects and analysis in pharmacology. New animal models could be evaluated by our method before they are used in drug discovery.

We applied the method to several cases of known animal model expression profiles and obtained some useful information to help drug research. We found that trichostatin A and some other HDACs could have very similar response across cell lines and species at gene expression level. Mouse hypoxia model could accurately mimic the human hypoxia, while mouse diabetes drug model might have some limitation. The transgenic mouse of Alzheimer was a useful model and we deeply analyzed the biological mechanisms of some drugs in this case. In addition, all the cases could provide some ideas for drug discovery and drug repositioning.

**Conclusions:**

We developed a new cross-species gene expression module comparison method to use animal models' expression data to analyse the effectiveness of animal models in drug research. Moreover, through data integration, our method could be applied for drug research, such as potential drug candidates, possible drug repositioning, side effects and information about pharmacology.

## Background

The use of animal models is essential in the study of many human disorders, especially in the occasions when human patients are inaccessible, or ethical issue prevents using human subjects in such studies. Animal models can greatly reduce the costs of research and thus they are available and affordable to a broad scientific community. Animal models have been proved to be important in the areas of chronic wasting diseases, i.e. Alzheimer [1-3], cancers [4-6], and new drug development [7-11]. A study found that animal models could predict human toxicity in 71% of the cases [12].

However, despite the advantages in employing animal models to study various human diseases, it has still been a challenging task in drug research to test thousands of compounds in animal models for searching a few promising candidates. Because important biological differences still exist between animal models and humans that could significantly impair drug discovery [11], although the models could usually recapitulate many of the key features in physiology. For example, mice do not own a true homologue of human interleukin-8 (IL-8), and presumably the function of this cytokine in mice is subsumed by other molecules. Thence, we cannot directly test IL-8 antagonists or agonists in murine systems [11]. In this regard, the scientific value of an animal model depends on how accurately it can mimic the human disease, and an assessment of the animal models' similarity to human disease state is requisite.

As a dynamic and continuous variable, expression changes with the developmental and physiological states. Furthermore, it is known that a gene's transcriptional response provides important clues to its function. Therefore, genes' expression profiles across species can be compared to determine the conservation and divergence of transcription. Microarrays have collected the necessary data to evaluate the transcriptomic fidelity of an animal model in terms of the similarity of expression with the human tissues.

Strand and his colleagues have proved that regional gene expressions of brains between human and mouse were conserved [13]. Miller et al. also undertook a brain-specific comparison of human and mouse transcription profiles [3], and in agreement with Strand's study, they found that both gene expression and the summation of gene co-expression relationships are generally well conserved. At the same time, they also identified some between-species differences that provided insight into human disease. However, whether orthologous gene pairs have the similar pattern of gene expression across species has been much discussed over the past two decades, but comparative analysis at the transcriptomic level has produced opposite conclusions [14,15]. Building on improved computational methods to correct such opposition, Chan et al. [16] compared multiple tissue-expression datasets across five vertebrate species: human, mouse, chicken, frog and pufferfish, and found the evidence of conserved expression in more than a third of unique orthologous genes. Consistent with Chan et al.' discovery, Zheng-Bradley et al. confirmed the conservation of gene expression at a greater degree [14,15] by carrying out a large scale comparison of global gene expression patterns in human and mouse. They proved that the global tissue-specific expression patterns of orthologous genes are considerably conserved in mouse and human, and the expression of groups of orthologous genes in each tissue co-varied, in both the tissue-specific gene and the house-keeping gene of two species.

In view of the above information, we proposed a novel approach to assess whether the animal models recapitulate the essential features of human diseases for drug research. The approach was based on the gene expression data of the response of function-known drugs from Connectivity Map (cMap) [17]. cMap had collected many microarrays corresponding to treatment of 164 different small molecules in different human cell lines. By comparing the gene expression signatures of drugs, diseased samples, and mutants, cMap was able to connect compounds, diseases, and genes through gene expression profiles. Considering the similarity of orthologous gene expression profiles across species, we first matched human and other animal species' genes using gene ortholog information in Roundup database [18], and then applied gene modularization technology to compare gene expression profiles, which was proposed by Li et al. [19]. We expected that this orthologous genes' similarity could provide a way to explore the ability of animal models to mimic diseases of the human bodies. When the connection of function-known drugs and the disease was established, we were able to infer whether these drugs were the right reagents to the corresponding disease and thus conclude the similarity between animal models and human's disease state. We also compared this gene modularization method with the distance method used by other researchers on cross-species analysis [14].

By applying the method to animal model expression profiles in several cases, lots of interesting information was obtained for drug research. We found that trichostatin A and some other HDACs could have very similar response across cell lines and species at gene expression level. Mouse hypoxia model could accurately mimic the human hypoxia, while mouse diabetes drug model might have much limitation in drug discovery. What's more, the transgenic mouse of Alzheimer was also an available model, and then we deeply analyzed the biological mechanisms of some drugs in this case. In addition, all the cases could provide some ideas for drug discovery and drug repositioning.

## Results

### Cross-species comparison of drug response at cell level

At first, we tested whether our cross-species method could find the similarity of drug responses across the species. From GEO, we downloaded 7 microarray data of mouse osteoblastic cells (MC3T3-E1) treated by Trichostatin A (TSA, an HDAC inhibitor), including three replicates of TSA treatment and four replicates of control (GEO: GDS3002). After performing one similarity search in the cMap database by our method, the top 10 chemicals with highest scores were presented in Table 1. The result of the distance comparison method was presented in Table 1.

**Table 1:** The top 10 instances obtained from gene modularization comparison method and distance comparison method in TSA case

**A**.	cMap ID	Molecule	GO counts	**B**.	cMap ID	Molecule
	1050	trichostatin A	56+		448	trichostatin A
	1072	trichostatin A	55+		909	HC toxin
	448	trichostatin A	54+		1072	trichostatin A
	1014	trichostatin A	53+		1000	vorinostat
	909	HC toxin	53+		1058	vorinostat
	981	trichostatin A	52+		981	trichostatin A
	1112	trichostatin A	50+		1050	trichostatin A
	992	trichostatin A	50+		332	trichostatin A
	331	trichostatin A	50+		331	trichostatin A
	332	trichostatin A	50+		1112	trichostatin A

The top 10 instances sharing the largest number of significantly affected GOMs with TSA-treated cells are listed in A and the top 10 instances resulted from distance comparison method are listed in B.

For detailed parameter settings: BP is chosen as GO mode, and the *P*-value for cutting off insignificantly matched or reverse-matched GO modules is 0.05. 'plus' indicates the number of GO terms positively correlated; 'minus' indicates the number of GO terms negatively correlated.

The results of our method and distance comparison method were consistent. TSA itself appeared many times. For the rest, Vorinostat, and HC toxin, in spite of distant structures, were all HDAC inhibitors [20-22]. Data in "GO counts" column (Table 1a) showed that these chemicals were almost fully-positively correlated with the query, consistent with the fact that they performed the similar function.

Although all the cells in cMap database were from human tumor cell lines and the query data were obtained from mouse osteoblastic cells (MC3T3-E1), the result indicated that the expression similarity existed between different cells and species when treated with HDAC inhibitors.

In 2009, Dudley et al. [23] evaluated 429 experiments, representing 238 diseases and 122 tissues from 8435 microarrays, and found evidences of a general, pathophysiological concordance between microarray experiments measuring the same disease in different tissues. Our result showed that microarrays of cell response to drugs which altered the cellular expression pattern could also have similarity across cell lines or species. The consistent result of our method and distance comparison method also hinted that cross-species gene expression analysis was practicable in the field of drug research.

### Exploring the effectiveness of mouse models of diseases and their relations with some drug molecules

Our approach could be used to determine whether the mouse model could be applied to preclinical drug screening and to identify potential novel drug or drug repositioning for certain diseases in the database. We tested three separate cases, *hypoxia, Diabetes drug *and *Alzheimer *by using gene expression profiles of mouse animal models.

#### Hypoxia

The response of mouse to hypoxia was derived from a study by Laifenfeld [24] in which mice received decreasing oxygen concentrations from 21% to 6% O_2 _for 30 minutes. Then, the mice remained at 6% O_2 _for another 120 minutes and the bone marrows were retrieved from the right humerus. We used 7 microarray assays (GEO: GSE17796) of bone marrow cells to run our test and the results were listed in Table 2a (our method) and Table 2b (distance comparison method).

**Table 2:** The top 10 instances obtained from gene modularization comparison method and distance comparison method in hypoxia case

**A**.	cMap ID	Molecule	GO counts	**B**.	cMap ID	Molecule
	595	resveratrol	25+		573	deferoxamine [INN]
	1015	genistein	22+		1068	thioridazine [INN]
	970	5230742	21+		418	haloperidol [INN]
	1068	thioridazine [INN]	20+		921	sirolimus [INN]
	573	deferoxamine [INN]	20+		1080	sirolimus [INN]
	1004	trifluoperazine [INN]	19+		906	calmidazolium
	991	tretinoin [INN]	18-		904	5109870
	871	ionomycin	18+		1075	fluphenazine [INN]
	921	sirolimus [INN]	17+		977	wortmannin
	444	clofibrate [INN]	17-		417	thioridazine [INN]

The top 10 instances sharing the largest number of significantly affected GOMs with bone marrow cells form hypoxia model are listed in A and the top 10 instances resulted from distance comparison method are listed in B.

Detailed parameter settings: GO mode: BP; *P*-value: < 0.05.

In Table 2a, nine in ten chemicals were reported to be associated with hypoxia and seven of the nine agents showed fully-positive correlation with the query profiles. Resveratrol was reported to inhibit the accumulation of hypoxia-inducible factor-1alpha and VEGF expression in human tongue squamous cell carcinoma and hepatoma cells [25], which seemed to have a protective mechanism in hypoxia mice. Genistein postconditioning had a protective effect on hypoxia/reoxygenation-induced injury in human gastric epithelial cells [26]. Thioridazine was a member of the class of phenothiazines that act, in part, by inhibiting respiration and lead to hypoxia [27]. Deferoxamine, a chelating agent capable of binding free iron, acted to simulate hypoxia by altering the iron status of hydroxylases [28]. The calmodulin inhibitor, Trifluoperazine, could suppress the hypoxic hyperpolarization [29]. Ionomycin was used to raise the intracellular level of calcium and calpain activity in rat proximal tubules in order to simulate the effects of hypoxia [30]. Sirolimus was an mTOR inhibitor that leads to the inhibition of the Hypoxia-inducible factor activity [31,32].

The remaining two of the nine agents showed negative correlation with the query profiles. Tretinoin (retinoic acid) stimulated erythropoietin gene transcription in embryonal carcinoma cells through the direct repeat of a steroid/thyroid hormone receptor response element half-site in the hypoxia-response enhancer element [33]. Clofibrate reduce hypoxia-inducible factor (HIF)-2alpha binding to the hypoxia-response element (HRE) [34]. In Table 2b, besides the molecules as mentioned above (deferoxamine, thioridazine and sirolimus), Haloperidol, Calmidazolium and Wortmannin were also reported to be associated with hypoxia [35-37].

Through these descriptions, we could see that the mouse model of the hypoxia was a good one to be used to observe the mechanism of hypoxia and help to discover drugs aiming to different targets or find side effects of some existing drugs in hypoxia. Moreover, our method could find some molecules (Tretinoin and Clofibrate) negatively correlated to hypoxia and they had a common feature: **effect on hypoxia-response element**. This result could not be obtained from the distance comparison method.

#### Diabetes drug

It had been reported that the mouse was not a reasonable animal model in the research of diabetes drug, because of its much lower AR expression level than that of human, which was probably insufficient to generate toxic by products [38].

We used our method to test if mouse models were suitable in diabetes drug study. We got microarray assays (GEO: GSE14888) of mouse 3T3-L1 adipocyte tissue cultures fed by metformin, then ran our method and the distance comparison method respectively, and presented the results in Table 3.

**Table 3:** The top 10 instances obtained from gene modularization comparison method and distance comparison method in Diabetes case

**A**.	cMap ID	Molecule	GO counts	**B**.	cMap ID	Molecule
	1069	15-delta prostaglandin J2	63+		941	rottlerin
	887	celastrol	54+		839	5224221
	893	pararosaniline	54+		1069	15-delta prostaglandin J2
	1011	15-delta prostaglandin J2	53+		978	pyrvinium
	839	5224221	53+		956	5224221
	956	5224221	51+		887	celastrol
	941	rottlerin	51+		893	pararosaniline
	1140	MG-132	46+		1140	MG-132
	1010	thioridazine [INN]	45+		1010	thioridazine [INN]
	976	5182598	44+		1011	15-delta prostaglandin J2

The top 10 instances sharing the largest number of significantly affected GOMs with adipocyte tissue fed by metformin are listed in A and the top 10 instances resulted from distance comparison method are listed in B.

Detailed parameter settings: GO mode: BP; *P*-value: < 0.05.

The results of our method and the distance comparison method were consistent. Among these molecules, only 15-delta prostaglandin J2 had some treatment relations with diabetes. It is a ligand of the adipocyte determination factor PPAR gamma [39,40]. Nevertheless, it was very confusing that Rottlerin was positive with metformin in our result, because Rottlerin could inhibit insulin-stimulated glucose transport in 3T3-L1 adipocytes by uncoupling mitochondrial oxidative phosphorylation [41]. Besides, Zhang et al suggested new possible applications (drug repositioning) for Celastrol, such as diabetes management [42].

Other molecules had no report of any relations with diabetes. Therefore, it was suggested that the mouse and human had some differences in the effect of metformin. However, it was possible to make use of mouse model to do drug research related to 15-delta prostaglandin J2, whose target was a nuclear receptor.

#### Alzheimer

Alzheimer disease (AD), the most common form of dementia, is incurable, degenerative and terminal. It has been advised that the mouse was not a good animal model for Alzheimer, because human and mouse's brain transcriptome had a large divergence in Alzheimer disease pathways [3]. But if the mouse was transgenic, would it become a suitable model?

The animal model we used here was a transgenic (TG) mouse expressing human APP695 and bearing the double Swedish and Indiana amyloid precursor protein (APP) mutations [1]. Six microarray assays (Hippocampus cells from two normal and four APP transgenic mice, GEO: GSE14499) were analyzed using our method and the distance comparison method. Top ten hits were presented (Table 4).

**Table 4:** The top 10 instances obtained from gene modularization comparison method and distance comparison method in Alzheimer case

**A**.	cMap ID	Molecule	GO counts	**B**.	cMap ID	Molecule
	415	nordihydroguaiaretic acid	25-		367	fulvestrant
	966	tretinoin [INN]	22-		447	tretinoin
	513	LY-294002	21-		3	metformin
	367	fulvestrant [INN]	20+		582	butein
	1048	alpha-estradiol	18-		419	chlorpromazine
	988	estradiol [INN]	15- 1+		942	prazosin
	953	monorden	16- 1+		970	5230742
	502	prazosin [INN]	17+		448	trichostatin A
	361	LY-294002	9- 8+		254	SC-58125
	866	ikarugamycin	16+		460	deferoxamine

The top 10 instances sharing the largest number of significantly affected GOMs with Alzheimer hippocampus cells are listed in A and the top 10 instances resulted from distance comparison method are listed in B.

For detailed parameter settings: GO mode: BP; *P*-value: < 0.05.

As the table showed, no molecules were found by the distance comparison method to have a treatment on Alzheimer. In contrast, six of the top ten results detected by our method were negatively related to Alzheimer, promising possible therapeutic functions. Nordihydroguaiaretic acid could break down pre-formed Alzheimer's β-amyloid fibrils in vitro [1]. Tretinoin (Retinoid) was relevant to many pathophysiological features of AD, including amyloid plaques, inflammation, immunological changes, cell death and regeneration processes, altered neurotransmission, and age-related changes [43]. It made sense that Nordihydroguaiaretic acid and Tretinoin both had many negative correlation GO modules and could resist AD. Estradiol and alpha-estradiol also prevented AD-associated inflammation with an increasing PPAR gamma expression [44]. Monorden, also known as radicicol, was a natural product binding to Hsp90 (Heat Shock Protein 90) and altering its function, while Hsp90 acted as a regulator of pathogenic changes that leaded to the neurodegenerative phenotype in AD [45]. LY-294002 held back the trafficking of APP and APP metabolites by inhibiting phosphatidylinositol 3-kinase (PI3K) [46].

Among the remaining molecules, Prazosin was a non-sedating generic medication used for hypertension and benign prostatic hypertrophy. It antagonizes NE effects at brain postsynaptic alpha-1 adreno receptors and new study said the prazosin improved patients' behavioural symptoms such as agitation/aggression in AD [47]. Fulvestrant was an interesting drug, known to block estrogen receptors [48]. It could also dissociate HSP90 and trigger its intracellular degradation [49]. Considering the positive connection between fulvestrant and Alzheimer, we could infer that estrogen pathway was more important than HSP90 pathway in AD (details described in the ***Discussion ***section). The last molecule, ikarugamycin, had no report of any relation with AD, but we thought it might also have a potential side effect to induce AD because of the positive correlated modules in our result.

Because almost all molecules were related with AD in the result of our method, we thought that the transgenic AD model was a feasible model of AD in humans.

## Discussion

**Figure 1:**
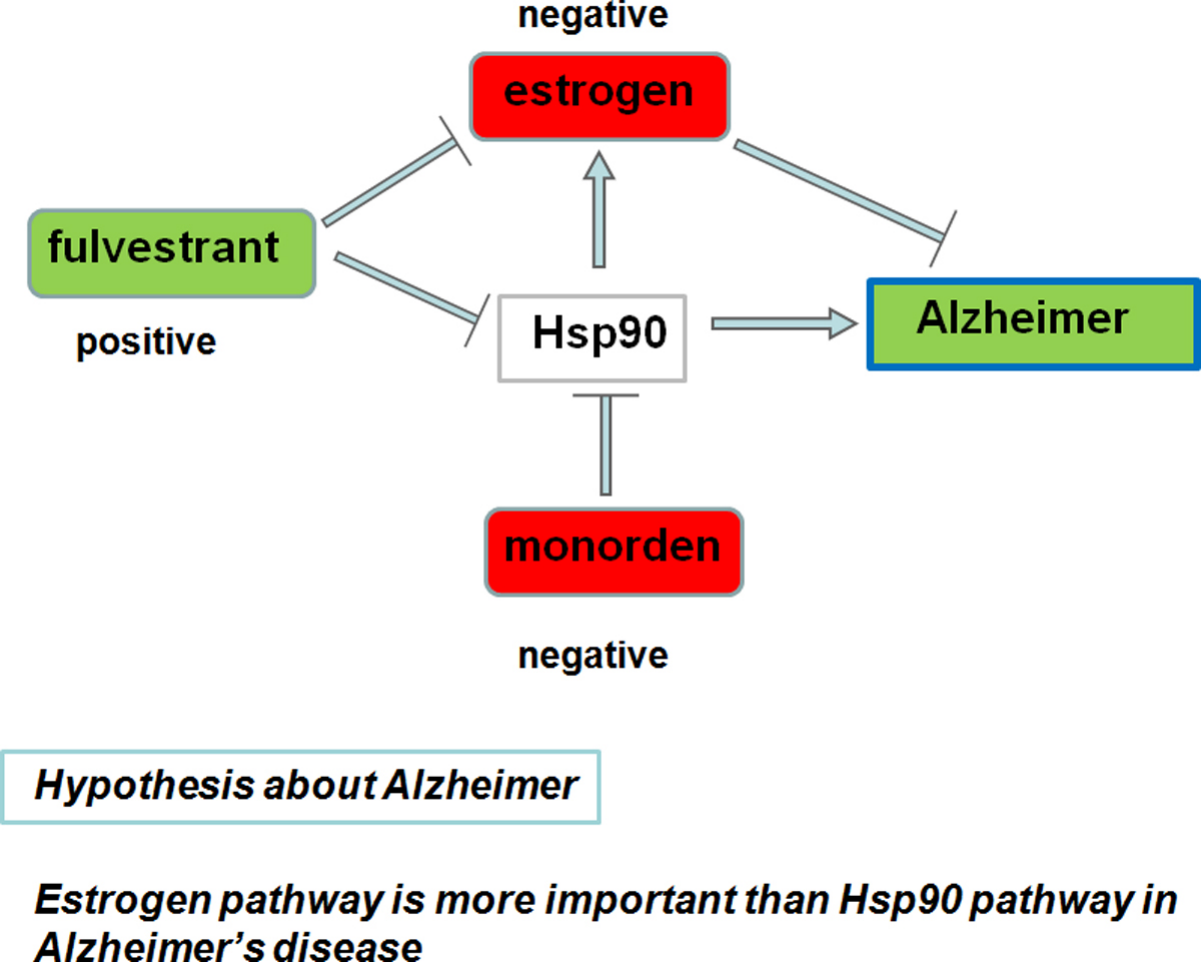
**In-depth analysis of the results of the Alzheimer disease case**. Alpha-estradiol and monorden are negatively connected with Alzheimer disease, while fulvestrant showed positive connection with Alzheimer. In this figure, we can see the fulvestrant-estrogen-Alzheimer pathway is more effective than the fulvestrant-Hsp90-Alzheimer pathway, so the positive connection of fulvestrant promise that estrogen pathway has more influence than the Hsp90 pathway in Alzheimer.

Since the transgenic animal model of AD was feasible for drug discovery, we further performed an in-depth analysis of the results of the AD case, especially for the three candidates: **fulvestrant, alpha-estradiol **and **monorden**. **Alpha-estradiol**, the predominant sex hormone presented in females, and monorden, a kind of HSP90 inhibitor, were both negatively connected with AD, while fulvestrant, both estrogen blocker and Hsp90 inhibitor, showed positive connection with AD (Figure 1).

Researchers had found that the molecular chaperone Hsp90 interacted with unliganded steroid hormone receptors (including estrogen receptor) and regulated their activity [50]. Thus, it is natural to infer that **monorden **might alleviate AD by acting Hsp90 receptor and regulating estrogen's pathway. However, according to our result, the exact GO modules of **alpha-estradiol **was mainly associated with lipid process (GO: 0008610, GO: 0006629), while monorden showed strong connection with sterol process (GO: 0016126, GO: 0016125) (see Additional file 1). The two different processes indicated that Hsp90 should have a second pathway to connect with AD.

Compared with GO categories of **alpha-estradiol **and **monoden**, **fulvestrant **had two additional categories, "phosphoglyceride metabolic process" (GO: 0006650) and "phospholipid biosynthetic process" (GO: 0008654), listed. Based on these, we hypothesize that estrogen might act the phospholipid pathway designed to alleviate AD. Literature mining helped us reveal that estrogen reduced the risk of AD by anti-Aβ (β-amyloid) [51]. Estrogen (17β-E2) accelerated βAPP trafficking and precludesmaximal Aβ generation within the TGN by modulating TGN phospholipid levels, particularly those of phosphatidylinositol. Therefore, **fulvestrant**, as estrogen blocker might activate phospholipid pathway to accelerate β-amyloid and aggravate AD. **Based on our analysis, we propose that: 1) estrogen receptor pathway acts through phospholipid to alleviate AD; 2) Hsp90 inhibitors have another pathway to alleviate AD rather than estrogen pathway; 3) Despite that both estrogen receptor and Hsp90 inhibitors are promising drug targets for AD, estrogen receptor is a much better candidate**.

The success of three cases between mouse models and human demonstrated that our cross-species analysis method was able to assess animal models' similarity to human's disease state. The main basis may be that orthologous genes were not only conserved at the sequence level and perform similar functions in different organisms, but also the corresponding gene expression patterns were conserved on a global level, especially between the human and mouse [15,16]. Our result that microarrays of cell response to molecules or drugs showed similarity across cell lines or tissues, to some extent, also explained why our approach was feasible to test mouse models. Nevertheless, as the diabetes drug case showed, it should be noticed that intrinsic differences always existed in normal and pathobiology states between species. [52]. Therefore, it was sometimes not appropriate for an animal model to mimic human diseases or drug response. Our method based on the analysis of the relationship of function-known drugs and human diseases utilizing microarray expression data performed well in both situations. Additionally, due to the introduction of GO annotations and the application of the statistical analysis, this cross-species method was able to provide bidirectional (both positive and negative) relationship between drugs and disease, and more clues about potential biological mechanisms. By contrast, the distance method seemed to be oversimplified and couldn't provide such information.

In this paper, we presented data only from mouse animal models, for the mouse was the main model for common human diseases. At present, the National Center for Biotechnology Information (NCBI) Gene Expression Omnibus (GEO) enrolled 1295 datasets on homo sapiens and 1069 datasets on Mus musculus. Another reason for using the mouse model was that the orthologous genes in mouse and human covered almost all genes in the cMap database.

Our cross-species analysis approach could also be extended to data from other cell lines, tissues, and human disease, which could be used to establish an animal model database instead of cMap. In addition, except for GO, other rules of gene partition such as KEGG were also good options. It was our primary goal to build extended references and additional gene modulation tools in the online service for biomedical research community.

## Conclusions

In the present work, we introduced a new cross-species gene expression module comparison method to make the most of animal expression data and analyze the effectiveness of animal models in drug research. Through exploring the relations between drug molecules and mouse disease models, our method was able to assess whether the corresponding model recapitulates the essential features of the human disease. If so, this model may be suitable for drug molecules screening or even to test novel therapies systematically. Moreover, through data integration, our method could mine some meaningful information for drug research, such as potential drug candidates, possible drug repositioning, side effects and information about pharmacology.

## Methods

### Data source and preprocessing

Drug molecule response data was downloaded from Connectivity Map (cMap) (http://www.broad.mit.edu/cmap). cMap is a collection of gene-expression profiles of cultured human cells treated with bioactive small molecules or drug molecules. The data set was composed of mRNA expression data for 164 distinct small molecules and corresponding vehicle controls applied to human cell lines (564 gene expression profiles in total, representing 453 individual instances). All the data was generated by means of Affymetrix GeneChip microarrays. We normalized every instance by ranking the gene expressions and stored them in our own database for comparison.

The data of animal models were downloaded from GEO. In TSA case, there were 7 microarray data of mouse osteoblastic cells (MC3T3-E1) treated by Trichostatin A, including three replicates of TSA treatment and four replicates of control (GEO: GDS3002). In hypoxia case, we used 7 microarray assays (GEO: GSE17796) of bone marrow cells. The response of mouse to hypoxia was derived from a study by Laifenfeld [24] in which mice received decreasing oxygen concentrations from 21% to 6% O_2 _for 30 minutes. Then, the mice remained at 6% O_2 _for another 120 minutes and the bone marrows were retrieved from the right humerus. In Diabetes drug case, we got microarray assays (GEO: GSE14888) of mouse 3T3-L1 adipocyte tissue cultures fed by metformin. In Alzheimer case, the animal model was transgenic (TG) mice expressing human APP695 and bearing the double Swedish and Indiana amyloid precursor protein (APP) mutations [1]. Six microarray assays (Hippocampus cells from two normal and four APP transgenic mice, GEO: GSE14499) were obtained.

### Orthologous gene matching

Orthologous gene conversion relied on the Roundup database [18] (http://roundup.hms.harvard.edu), a large-scale database of orthologs. The orthologs were computed by the Reciprocal Smallest Distance (RSD) algorithm, which was developed by Wall et al. [53]. For human and mouse, about 13264 genes were selected by RSD algorithm. These genes covered almost all genes in the small molecule database of cMap.

### Gene modularization comparison method

**Figure 2:**
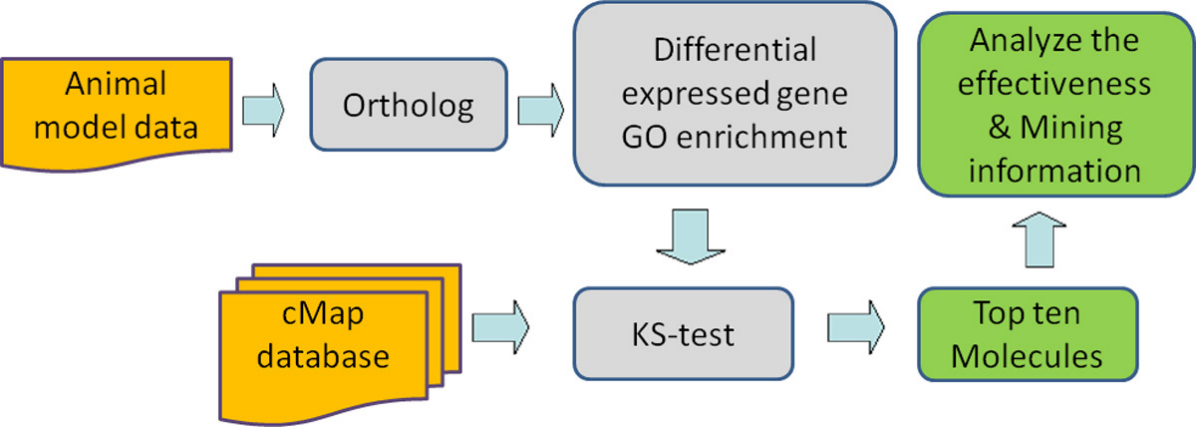
**Workflow of our analysis method**. The cMap database of chemical interventions was used for search. Given an animal model query profile with gene id and expression value (treated/control), every animal gene id was converted to orthologous human gene id. Through differential expressed gene GO enrichment and KS-test with cMap database, a result was returned with a table of top 10 molecules which were most similar to that of the query profile, and their similar GO modules, positive or negative.

The processes of our method are depicted in Figure 2. After ortholog matching on the gene expression data of animal model, 1.5-fold change was used as default threshold for differential expression (2-fold change, 1.3-fold change or t-test could also be used), and then hyper geometric test was performed in every Gene Ontology Module (GOM). We chose Gene Ontology Module as our modularization reference, because it was the most widely used in exploring biological features of genes with respect to their molecular functions, biological processes as well as cellular components.

GOMs were selected, when p-values from hyper geometric test were smaller than 0.05. Based on each selected GOMs, the expression pattern similarity between the animal model data and the chemicals data in the cMap database was calculated.

The algorithm was derived from Kolmogorov-Smirnov statistics (KS-test), which was called connectivity score in Lamb et al.'s work [17]. But Lamb et al. applied the algorithm on the whole profile and we applied it in every GOM. The KS score indicated the similarity of two samples. For each GOM, it showed genes that had the same or reverse pattern of expression between the query and reference chemicals. If the KS score was positive in a certain GOM, the query and reference chemicals would have similar pattern of expression in this GOM, and vice versa. P-value was also calculated to indicate significance of the comparison [19]. Similarly, only GOMs with p-value < 0.05 would be selected.

The result of performing one similarity search was a table, whose each column represented a chemical in reference library and each row represented a GOM. The value in each grid was the KS score or p-value (the p-value of comparison, not the p-value of hyper geometric test) of the query and reference chemicals in certain GOM. The top 10 reference chemicals which had the most similar GOM numbers were selected for each analysis.

### Distance comparison method

As a control to our method, we also used distance method to perform a cross-species analysis. The distance method has been used by other researchers in the cross-species analysis, where euclidean distances were computed to cluster the similar samples [13]. But in this study we applied absolute distances to show the similarity between the gene expression data from animal model and human, in the case that all the gene expression data in the cMap database was given ranking values.

First, orthologous genes matching and differential expression analysis were done on the gene expression data of animal models. Then the differential expressed genes were ranked, similar to the corresponding genes of each instance in the cMap. Absolute distances were calculated between the animal model and each instance by

$$\left|x_{1}-y_{1}\right|+\ldots +\left|x_{k}-y_{k}\right|$$

where *k *means the number of genes and *x *and *y *are animal and instances samples, respectively. The top 10 instances which had the smallest distance values were selected.

## Competing interests

The authors declare that they have no competing interests.

## Authors' contributions

SY performed design. SY and LZ statistical analyses for the study and drafted the manuscript. YL, CL and CM participated in the design of the study and provided guidance. YXL, PH and XL conceived the study, and finalized the organization and contents of the manuscript. All authors approved the final manuscript.

## Supplementary Material

Additional file 1 **All GOMs of alpha-estradiol, monoden and fulvestrant in our result of Alzheimer case**. The yellow GO modules are associated with lipid or sterol process, indicating these modules involved Alzheimer disease may act through these process. The green GO modules are only enriched in the result of fulvestrant. Click here for file
